# Giving substance to ‘the best interpretation of will and preferences’

**DOI:** 10.1016/j.ijlp.2018.12.001

**Published:** 2019

**Authors:** Paul Skowron

**Affiliations:** York Law School, University of York, UK

**Keywords:** Best interpretation, Will and preferences, UNCRPD, Convention on the Rights of Persons with Disabilities, General Comment no. 1, Best interests

## Abstract

In General Comment No. 1, the UN Committee on the Rights of Persons with Disabilities calls for ‘the best interpretation of will and preferences’ to replace best interests determinations in decision-making law, but it has given little guidance on the content of this new standard. As a result, ‘best interpretation’ is sometimes treated as synonymous with ‘true interpretation’. On this reading, ‘the best interpretation of will and preferences’ is just whatever interpretation most accurately represents the interpreted person's will and preferences.

This article shows that the conflation of the word ‘best’ with the word ‘true’ must be avoided. Interpretative processes contribute to changes in the interpreted person, including changes in their will and preferences. There are both supportive and abusive forms of these contributions, but conflating ‘best interpretation’ with ‘true interpretation’ removes both from view. An alternative reading of ‘best interpretation’ should therefore be preferred: one that requires the process of interpretation to be responsive to both truth and the detailed substantive rights found in the UN Convention on the Rights of Persons with Disabilities.

## 1 Introduction

Article 12 of the UN Convention on the Rights of Persons with Disabilities (‘the UNCRPD') guarantees people with disabilities ‘equal recognition before the law’.^1^ It also establishes a Committee on the Rights of Persons with Disabilities (Article 34). State Parties make regular reports to this Committee about their compliance with their Convention obligations (Article 35), and it makes ‘suggestions and general recommendations' in response (Article 36). In these responses, the Committee has frequently observed that people with mental disabilities are still subject to discriminatory guardianship regimes that remove or impair their ability to exercise legal rights (UN Committee on the Rights of Persons with Disabilities, 2011).^2^ Beyond that, it has observed such a ‘general misunderstanding’ of Article 12 that it ‘has found it imperative to provide further guidance’ (UN Committee on the Rights of Persons with Disabilities, 2014: para. 3) in the form of General Comment No. 1 (‘GC1’). This guidance, however, is necessarily written at a high level of generality, so it neither settles all of the controversies that existed during the Convention's drafting process (Dhanda, 2007) nor fully develops all of the claims that it makes about the ‘normative content of Article 12’ (UN Committee on the Rights of Persons with Disabilities, 2014: part II).

This article addresses one claim made in GC1: the statement that the ‘best interpretation of will and preferences’ must replace ‘best interests’ decisions for adults (para. 21). This claim is controversial. Many states use variations on best interests systems, and some authors believe that it is possible to modify such systems to make them UNCRPD-compliant (Martin et al., 2014); but it can seem that the Comment entirely rules out this approach. The idea of a simple either/or choice here, however, can distract from the level of complexity that exists on both sides of the issue. On one hand, the operation of best interests systems is more complex than either statutory language or judicial statements usually imply (Skowron, 2018). On the other hand, without a relatively precise account of what ‘the best interpretation of will and preferences’ requires, any comparison between it and ‘best interests’ is premature. This article addresses the latter point. It is a step towards developing a clear account of best interpretation.

It is especially important to clarify what ‘best interpretation’ requires because the phrase is ambiguous. The ‘best interpretation’ of a person's will and preferences could refer to two different things. It might refer to the best statement of a person's will and preferences, the *outcome* of a process of interpretation; but it might also refer to the best method of interpreting a person, the *process* of interpretation itself. When it comes to understanding ‘best interpretation’, it is essential to address this ambiguity. To evaluate any set of practices against a standard, it is necessary to first know where the standard applies. State Parties must know what to measure if they are to assess their compliance with GC1.

This article shows that there is an overriding reason to read the Comment as demanding a particular process of interpretation, not as demanding a particular type of outcome: outcome measures pay insufficient attention to the ways that people are changed and preferences are brought into existence by acts of interpretation. This matters for two reasons. First, some interpretative processes that change a person's preferences, such as offering them new options, may be acceptable, but others, such as ones that undermine their confidence, will not be; and it is important to be able distinguish between the two. Second, the way that a person's preferences change during the process of being interpreted can be an expression of their agency, but outcome measures subtly misattribute this agency to the interpreter.

Section two shows that although there is some evidence that the Committee intended ‘best interpretation’ to apply primarily to the process of interpretation, this is ambiguous. It also, however, makes a further important point. ‘Best interpretation’ in the Comment is sometimes read as a special procedure *only* to be used when it is not ‘practicable to determine the will and preferences of an individual’ (para. 21), but the section shows that GC1 instead calls for best interpretation *even* when it is not ‘practicable to determine the will and preferences of an individual’. In other words, this part of GC1 calls for inclusion, not for a procedure to be applied only in certain circumstances.

Section three raises the most natural, and common in the literature, outcome reading of ‘best interpretation’. Called here the ‘epistemic reading’, this is the idea that the ‘best interpretation’ of a person's will and preferences is simply whichever interpretation most accurately reflects certain facts about the person. In other words, this reading assumes that the word ‘best’ in ‘best interpretation’ is synonymous with the word ‘true’. Section four then compares the epistemic reading to real processes of interpretation. It finds the reading wanting, for it occludes from view the ways that processes of interpretation change a person, create new preferences, and are themselves an arena for the person to exercise agency.

Section five proposes a process reading as an alternative to the epistemic reading. Its central point is that, when evaluating the ways that interpretation can change a person, the UNCRPD has not left a void. It provides a great deal of normative guidance. The reading of GC1 proposed here is new, but the underlying picture of interpretation is not, and it may be familiar to psychiatrists in particular. As Jaspers pointed out, psychiatrists must practice on both sides of a ‘radical difference’ between ‘scientific uncommittedness’ and ‘the doctor's participation in the fate of his client’: they must ‘surrender to the facts’ yet be ‘engaged' in a way that will change those facts even as they assess them (1959/1997: p. 677). This article makes a similar point about anyone who interprets another person in the hope of enabling them to exercise their legal rights.

## 2 Disambiguating ‘the best interpretation of will and preferences’

### 2.1 Structure of this section

This section performs two main tasks. It shows that the phrase ‘best interpretation of will and preferences’ is ambiguous: the words ‘best interpretation’ could refer to either a process of interpretation or to the outcome of that process. It also shows that, whichever reading is favoured, the scope of ‘best interpretation’ is necessarily wider than is sometimes asserted.

GC1 states that:

Where, after significant efforts have been made, it is not practicable to determine the will and preferences of an individual, the ‘best interpretation of will and preferences’ must replace the ‘best interests’ determinations. This respects the rights, will and preferences of the individual (para. 21).

This passage contains three relevant ambiguities. Do the words ‘best interpretation’ apply primarily to a process of interpretation or merely to its outcome? Similarly, although less importantly, does the word ‘determine’ apply to a process of determination or merely to its outcome? Finally, what is the role of ‘best interpretation’ when it *is* ‘practicable to determine’ a person's will and preferences? The next two subsections address the first question, and subsection 2.4 addresses the final question. The ambiguity in the word ‘determine’ is less critical, so it is addressed here.

GC1 requires ‘best interpretation’ if ‘it is not practicable to determine the will and preferences of an individual’ (para. 21). ‘Determine’ could, like ‘interpretation’, plausibly refer to either a process of investigation or to its outcome. In other words, the Comment could be requiring ‘best interpretation’ either when it is impossible to begin a process of determining a person's will and preferences or when such a process has, for some reason, failed.^3^ For the purposes of this article, an inclusive definition can be used. It will be assumed that GC1 requires ‘best interpretation’ both when it is not possible to begin a process of determining a person's will and preferences and when such a process has, for whatever reason, failed.

### 2.2 The process reading of ‘best interpretation’

At this stage, it is necessary to be a little clearer about the difference between the process and outcome readings of ‘best interpretation’. It might be thought that this distinction cannot be maintained and that the best outcome is only ever reached by the best process. On the level of interpreting one individual this may be true,^4^ but that is not the level on which GC1 operates. Instead, it sets the ‘paradigm’ within which all such individual interpretations should occur (para. 21). In this context, is important to settle the question of priority: whether the ‘best outcome’ is ‘best’ because it is the product of the ‘best process’ or the ‘best process’ is best because it produces the ‘best outcome’. This is important because the two possibilities yield entirely different systems, as sections three to five show. On the process reading, the ‘best interpretation’ is the one that follows the best process.

The broader context gives the process reading an immediate plausibility. GC1 is intended to provide ‘guidance’ to State Parties (para. 2). It is concerned with what States should be doing to comply with the Convention, and any ‘doing’ is a process. The narrower context, however, presents the process reading with an immediate difficulty. How does one ‘interpret’ a thing that one cannot ‘determine’? For someone to interpret their dream, they must remember it. For an actor to interpret the role of Hamlet, they must be familiar with Shakespeare's script. Interpretation implies familiarity with the thing interpreted. GC1, however, requires that a person's will and preferences be interpreted exactly when they cannot be determined; and this problem cuts across both possible meanings of ‘determine’. Whether ‘it is not practicable to determine’ is taken to refer to a process of determination that has failed or to one that could not even be undertaken, the same problem presents itself. The Comment requires the interpretation of an unknown. Whether the investigation fails or it cannot even begin, the interpreter will not know the ‘will and preferences of an individual’ in order to interpret them.

The process reading of GC1 can be rescued from this problem by paying attention to the Comment's drafting history. ‘The best interpretation of will and preferences’ was not in the original draft (UN Committee on the Rights of Persons with Disabilities, 2013). It was introduced in response to a submission made by The Canadian Association of Community Living (2014). This submission, however, simply suggests that ‘such a test would recognize that will and preference cannot always be interpreted with certainty, but that there are always better interpretations than others’ (2014: p. 5). Unlike GC1, this does not require the ‘interpretation’ of what cannot be ‘determined'. Instead, it simply recognizes that interpretation can be difficult, but affirms that it always possible. If it is assumed that the Committee intended to incorporate this submission into GC1, and did not intend the incoherence of requiring the interpretation of an unknown, then the process reading can be preserved. Doing so, however, has two costs. First, abandoning a hard distinction between ‘determining’ will and preferences and ‘interpreting’ them significantly expands the scope of ‘best interpretation’. This is discussed in subsection 2.4. Second, it requires adding a significant gloss to the words of the Comment, and this should be avoided if possible. If the outcome reading could bypass these difficulties, then this would be a reason to favour it. The next subsection shows that it cannot.

### 2.3 The outcome reading of ‘best interpretation’

In the outcome reading of ‘best interpretation’, the phrase does not refer to a process of interpretation but to the product of such a process. In other words, on this reading the ‘best interpretation’ is something like the ‘best understanding’ of the person's will and preferences, not the process for reaching such an understanding. As the process reading requires adding a significant gloss to the words of GC1, the outcome reading should be considered; but it, too, faces a difficult question. For the outcome reading, the difficult question is ‘how is the outcome arrived at?’

A possible answer to this question appears in the academic literature. Arstein-Kerslake and Flynn (2016: p. 485) recognize that sometimes a decision ‘needs to made’ even when a person's preferences about that particular decision are ‘unknowable’; but assert that even in these cases ‘some understanding of an individual's values, views and beliefs’ can be achieved. In other words, when there are difficulties inferring a person's will and preferences about a particular matter, attention should be paid to their longer-lasting or more general beliefs, values, and desires. This distinction can be read into GC1 itself by effectively adding two words:

Where, after significant efforts have been made, it is not practicable to determine the *particular* will and preferences of an individual, the “best interpretation of *overall* will and preferences” must replace the “best interests” determination (para. 21, as modified).

In this context, ‘particular’ refers to an unknown will or preference in question in any given situation, and ‘overall’ refers to the wider array of all the person's known will and preferences. This rescues GC1 from the problem, discussed in the previous subsection, of requiring the interpretation of an unknown because it makes the two appearances of the phrase ‘will and preferences’ refer to different things.^5^ In doing so, however, it also puts indirect pressure on the apparent dichotomy between processes of ‘determining’ and ‘interpreting’.

The distinction between ‘particular’ and ‘overall’ will and preferences puts pressure on the distinction between ‘interpreting’ and ‘determining’ because of how people determine one another's particular will or preferences in everyday life. They partially do so by reference to the person's overall will and preferences. Interpreting a person's overall will and preferences is always, to some extent, required to understand the person's particular preferences. Take, for instance, the common English phrase ‘do what whatever you like’. Its meaning is entirely context-dependent. A boss might say it to indicate that they trust a subordinate to be self-directed. A patient might say it to indicate that they do not trust a nurse, but feel coerced. A lover might even say it to indicate that they want their partner to stop merely doing whatever they like.^6^ The particular preference that the phrase discloses is only revealed by reference to the context, and the speaker's overall will and preferences are, as in the examples, often the most important part of that context. There is either a dialogue between interpretation of the whole and interpretation of the part, or there is simply no interpretation. As Taylor (1985: p. 18) writes:

We are trying to establish a reading for the whole text, and for this we appeal to readings of its partial expressions; and yet because we are dealing with meaning, with making sense, where expressions only make sense or not in relation to others, the readings of partial expressions depend on those of others, and ultimately of the whole.

This constant interplay between interpretation of the part and whole collapses the distinction between ‘determining’ a person's will and preferences and ‘interpreting’ them in much the same way that attention to the original submission by the Canadian Association of Community Living did in subsection 2.2. On examination, it becomes clear that ‘best interpretation’ is not a special procedure for when something goes wrong. It is a call to include people with disabilities in an everyday process, even when this is difficult. In other words, it directs attention to parts of interpretation that can plausibly continue even when there are severe communication problems. This has two consequences. First, if GC1's apparent dichotomy between ‘interpreting’ and ‘determining’ breaks down on either a process or outcome reading, then it does not provide any reason to favour one reading over the other. Second, as the dichotomy breaks down on both readings, it must be discarded, and this has consequences for the scope of ‘best interpretation’.

### 2.4 The scope of ‘the best interpretation of will and preferences’

The previous two subsections show that whether the process or outcome reading of ‘best interpretation’ is taken, a strict line between ‘determining’ and ‘interpreting’ a person's will and preferences cannot be maintained. This is directly relevant to another ambiguity in GC1. The Comment states that ‘where …it is not practicable to determine’ will and preferences, then ‘best interpretation’ applies. This is open to two readings. It might mean that best interpretation is required *only* where it is not practicable to determine will and preferences, or it might mean that best interpretation is required *even* where it is not practicable to determine will and preferences. The former maintains a strict line between ‘determining’ and ‘interpreting’, and demands a special procedure for when determining ‘will and preferences’ fails. The latter collapses that strict line, and demands that everyday practices of ‘interpreting’ continue even when ‘determining’ fails. As there are independent grounds for believing that a strict line between ‘determining’ and ‘interpreting’ cannot be maintained, the latter reading should preferred.

There are also contextual reasons for reading GC1 as demanding ‘best interpretation’ *even* when it is not practicable, rather than *only* when it is not practicable, to determine a person's will and preferences. The Comment introduces standards based on ‘will and preferences’ that apply to all situations. It states that one characteristic of ‘substitute decision-making’ regimes, which it requires to be abolished (para. 28), is that decisions are ‘based on what is believed to be in the objective “best interests” of the person concerned, as opposed to being based on the person's own will and preferences’ (para. 27).^7^ Similarly, GC1 requires that ‘all forms of support in the exercise of legal capacity …must be based on the will and preference of the person, not on what is perceived as being in his or her objective best interests’ (para 29(b)). These passages apply a ‘will and preferences’ standard; but make no distinction between when will and preferences can and cannot be determined. Furthermore, a reading that requires ‘best interpretation’ *only* when will and preferences cannot be determined would draw a legal line between some people with disabilities and the rest of the population on the basis of a distinction that, as already shown, cannot be sustained. The UNCRPD makes ‘full and effective participation and inclusion in society’ and ‘respect for difference and acceptance of persons with disabilities as part of human diversity and humanity’ general principles for its interpretation (Article 3), so such a step should be avoided.

Reading the call for ‘best interpretation’ as a call to include people with disabilities instead of a demand for a special procedure is significant for two reasons. First, it massively extends the scope of ‘best interpretation’. If best interpretation is demanded *even* where it is not practicable to determine will and preferences, then it is also demanded when it is practicable to determine will and preferences. In other words, if will and preferences are relevant, then ‘best interpretation’ is relevant. Due to GC1's ambiguity, this point is not always appreciated. For instance, Donnelly treats ‘best interpretation’ as the ‘fallback position’ (2016: p. 326). Second, if the demand for ‘best interpretation’ is a call to include people in everyday processes of interpretation, then close attention to those everyday practices is required. Later sections show that attention to these practices provides a strong reason to reject the most popular and natural outcome reading of ‘best interpretation’: the epistemic reading.

## 3 The epistemic reading of ‘best interpretation’

The previous section makes the initial distinction between process and outcome readings of ‘best interpretation’. Neither reading, however, is monolithic. In particular, different outcome readings are possible simply because it is possible to characterise different types of outcome as ‘best’. This section introduces the ‘epistemic reading’. This is the most important outcome reading, both because it is one of the most natural ways to read GC1 and because it is the most common in the secondary literature. This claim that it is the most common outcome reading, however, must be treated with care. The same ambiguity between process and outcome readings that appears in the Comment is endemic in the secondary literature. As a consequence, this section contains examples of authors relying on the epistemic reading, but it is seldom clear whether or not those authors would consciously endorse it on reflection. To some extent, that issue is irrelevant. Section four shows that the epistemic reading blocks from view the ways that processes of interpretation change a person, create new preferences, and are themselves an arena for someone to exercise agency. These risks exist whether or not the person reading ‘best interpretation’ in this way would reflectively endorse it.

The outcome that the epistemic reading treats as ‘best’ is, as its name suggests, a state of knowledge. In particular, it is a state of knowledge in the mind of the interpreter. For the purposes of this article, the epistemic reading is any reading that assumes or asserts that the ‘best’ interpretation is simply whichever interpretation results in the interpreter having the most accurate understanding of the interpreted person's will and preferences at the end of the process of interpretation. Effectively, this reading substitutes the word ‘true’ for the word ‘best’. This section shows that the epistemic reading is plausibly suggested by GC1 and common in the secondary literature.

A contextual cue in GC1 makes the epistemic reading plausible. The Comment repeatedly contrasts ‘will and preferences’ with ‘best interests’: twice in the paragraph that introduces ‘best interpretation’ (para. 21), and then again in two other paragraphs (paras. 27, 29(b)). Furthermore, this contrast is reinforced by another. Substitute decision-making, which is partially defined in terms of ‘best interests’ (para. 27), is also repeatedly contrasted with respect for ‘will and preferences’ (paras. 17, 26). It easy to generalise these contrasts into a binary in which a person's ‘will and preferences’ are a fact about them, their ‘best interests’ are a fact about the world, and there is no necessary connection between the two. If the binary is taken as complete in this way, then the epistemic reading emerges naturally. If the content of ‘will and preferences’ is taken to be only facts about the person, then it easy to assume that ‘the best interpretation of will and preferences’ is simply the one that discovers those facts as accurately as possible. Although section five shows that this reading is not required by GC1, it is easy to see how it is emerges from it.

The plausibility of the epistemic reading is bolstered by its appearance in an academic article by Flynn and Arstein-Kerslake that was influential on the drafting of GC1*.*^8^ Although it does not use the ‘best interpretation’ terminology, this article argues that even interpretation that amounts to a supporter trying to ‘imagine what the person's will and preferences might be’ must be completely distinguished from ‘whether or not the decision is a good one’ (Flynn and Arstein-Kerslake, 2014: pp. 96–97). It might seem strange to attribute the epistemic reading to an article that talks so frankly about ‘imagining’ a person's preferences. Nevertheless, due to a distinction discussed in section two, it is accurate to do so. Flynn and Arstein-Kerslake distinguish between a person's will or preference about a particular matter and their overall will and preferences, and they give the latter priority to a startling degree. For instance, they write that ‘a verbal expression in one instance may not necessarily represent the true will and preferences of an individual’ (Flynn and Arstein-Kerslake, 2014: p. 98). This allows them to admit that a supporter might be ‘imagining’ the particular preference that is in question, without treating the interpretation of the person's overall will and preferences as anything other than a matter of discovering the truth about a state of affairs. Indeed, they repeatedly use the phrase ‘*true* will and preferences’. An epistemic reading is also implied by other parts of their analysis. For instance, they posit a complete separation between the person's ‘supreme’ will and preferences and anybody else's evaluation of ‘the resulting decisions as “good” or “bad”’ (2014: p. 98). This strongly suggests that the ‘best’ process of interpreting the person's will and preferences is simply whichever one results in a true account of certain facts about the individual.

Flynn and Arstein-Kerslake are not the only authors to distinguish between a person's preferences about a particular matter and their longer-lasting or more general beliefs and desires. Szmukler uses the terminology of the UNCRPD itself to distinguish between a person's currently expressed ‘preferences’ and the ‘will’, which is ‘founded on a person's deeply held, reasonably stable and reasonably coherent personal values’ (Szmukler, 2017: p. 94). His epistemic reading is subtle, and this makes it valuable for illustrating an important distinction. Szmukler is attentive to the social and normative character of interpretation (2017: pp. 95–96).^9^ His epistemology is not naïve. He, however, treats interpretation as a purely epistemic task. He does not discuss the ways that processes of interpretation themselves change the person and their will and preferences. Instead, his entire discussion is of the interpreter's understanding of the person's mental states. Even the section on ‘changes in beliefs and values’ is written entirely in this mode (2017: p. 96). As a consequence, although Szmukler rightly acknowledges that interpretation has an inescapable normative component, he does not consider the full array of normative standards that any process of interpretation must be held to.

More sceptical responses to GC1 have also engaged primarily with the epistemic reading. For instance, Series (2015: p. 88) observes that ‘it is not immediately obvious that a claim to know a person's *true* will and preferences better than they do is a preferable basis for coercive intervention …than mental incapacity and best interests’. Indeed, several years before GC1 was drafted, Ward (2011: p. 25) warned of the dangers of ‘a decision more truly reflective of the views and values of the supporter, masquerading as the supported decision of the adult’. There is an obvious conflict between this view and that of Flynn and Arstein-Kerslake. They treat the determination of a person's preferences as the only process that can confer legitimacy on an action done in that person's name, but Ward treats the same process as inherently dangerous. Behind the surface conflict, however, there is a deeper agreement. The epistemic reading frames the entire debate. It is assumed that either a person's preferences, existing separate to the process of interpretation, can be known, in which case interpretation is legitimate; or they cannot be known, possibly because they do not exist, in which case interpretation is not legitimate. The more sceptical authors sometimes make this framing explicit. For instance, Donnelly says ‘the best interpretation standard refuses to acknowledge that there are things that we do not, and cannot, know. It forgoes epistemic humility and assumes levels of knowledge (and justifications for actions) in situations where they do not exist’ (2016: p. 327). As a critique of the epistemic reading, her point has considerable force. If, however, the ‘best’ of ‘best interpretation’ is not reduced to ‘true’ alone, these criticisms may miss their mark.

## 4 Everyday interpretation

### 4.1 The role interpretive presumptions

Subsection 2.3 concluded that the demand for ‘best interpretation’ is a call to include people with disabilities in everyday interpretative practices, not a demand for a special procedure to be developed only for when will and preferences cannot be determined. If this is so, then it is necessary to pay attention to the implications that everyday interpretative practices have for supporting a person's legal capacity. This section shows that these practices present a serious challenge to the epistemic reading. Everyday interpretative acts change the interpreted person and their will and preferences; and do so in both acceptable ways, which create new opportunities for the person, and unacceptable ones, which constrain the person. As the epistemic reading only evaluates the state of knowledge that an interpreter has at the end of a process of interpretation, it is incapable of distinguishing between these good and bad effects of interpretative acts. This subsection draws attention to a pervasive and important feature of everyday interpretation: the use of presumptions. The next subsection shows the challenge that this practice presents to the epistemic reading, and the final subsection addresses a possible objection to this analysis.

The UNCRPD literature to date almost universally contains examples of people with relatively severe disabilities being interpreted by professionals who are tacitly assumed to have no such disabilities. There is a place for such examples; but if treated as the entire field, then they are dangerous. They focus too much attention on the, real or imagined, ways that interpreting people with certain disabilities is different to ‘normal’ without first providing any account of how ‘normal’ interpretation operates. It is necessary to go slower. For this reason, the following example of an act of interpretation does not mention disabilities, although the participants may have almost any disability or none. In this example, Steve and Janet are shopping. Steve says ‘I want to go home’. To understand the words, Janet will make a number of interpretative presumptions. For instance, she will probably presume that Steve is using the words as people usually do, that he is referring to their current situation, and that he desires his preference go home to guide their behaviour.^10^

The literature on will and preferences often mentions presumptions, but it treats them as something to be used as a last resort for when the determination of will and preferences fails (Flynn and Arstein-Kerslake 2014: p. 98; Gooding, 2015: p. 55; Donnelly, 2016: p. 326)). This is a consequence of focussing on difficult examples without first having a clear account of the everyday interpretative practices that provide the wider context. This ‘last resort’ usage might be one function of presumptions, but it is a relatively marginal one. As the example shows, presumptions are not only present when interpretation ‘fails’. They are necessary for interpretation to occur in the first place. This alone renders them worthy of closer attention.

The form of presumptions can be summarised as follows: ‘in conditions *C*, take *P* to be true, unless *D'* (adapted from Resher, 2006: p*.* 33).^11^ This is best analysed from the outside in, examining *C* and *D* before *P.* The conditions (*C*) in which interpretative presumptions apply can be extremely broad. For instance, the presumption that someone understands a word that they use applies to almost all spoken communication, although it is sometimes rebutted. This latter point, presumptions can be rebutted, is intrinsic to their form. This is the meaning of ‘unless *D*'. *D*, however, does not refer merely to contradictory evidence. It refers to contradictory evidence *on interpretation*; and that secondary interpretation, too, will rely on presumptions.

In the example, Janet might respond to Steve saying that he wants to go home by replying ‘but we just got here’, and Steve might then say ‘I don't mean the house. I mean I'm tired of London. I want to go back to Scotland'. If so, Janet's presumption that he was referring to their immediate situation will be rebutted. It is only rebutted, however, if she presumes, among other things, that a later clarification should have priority over the original statement it clarifies. This new presumption could, itself, be rebutted if, for instance, Janet was visibly hurt by Steve's initial statement. Then, especially if Steve looks flustered, an observer might suspect that Steve really does want to go back to the house and that his comment about Scotland is just a panicked attempt to cover this up. Rebutting a presumption does not reduce the number of presumptions operating within a process of interpretation. It increases it, by drawing on other presumptions. Interpretative presumptions are utterly pervasive in every act of interpretation. This is important because, as the next subsection shows, their operation is not compatible with the epistemic reading.

### 4.2 Interpretation changes a person and their will and preferences

In the example, Steve's act of clarification – ‘I don't mean the house. I mean I'm tired of London…’ – highlights an important detail. A person's will and preferences are not inert objects. They contribute to their own interpretation. Steve's desire to return to Scotland motivated him to clarify when he was misinterpreted. Conversely, other preferences, particularly those that the person feels shame about, might hinder their own interpretation. If, however, a person, and their will and preferences, might be active during interpretative acts, then this puts pressure on the epistemic reading. If interpretative acts provide opportunities for the interpreted person to express preferences, then these expressive opportunities may have a value that will not be captured by any view that assesses only the interpreter's state of knowledge. For example, Steve may feel a sense of relief at getting a worry off his chest. An interpretative act that allows him to do so may be preferable to one that does not, regardless of its effect on the interpreter's state of knowledge.

The problems for the epistemic reading are, however, far more severe than this. Interpretative acts do not just create opportunities for the person to express themselves. They can also change a person's will and preferences. A person's will and preferences change in response to their environment; and, because humans are extremely social creatures, they particularly change in response to interpretation by others. This self-interpreting, responsive aspect of preferences is fatal to the epistemic reading. If interpretation sometimes changes the person's preferences, then it is important to evaluate how it does so; but the epistemic reading only evaluates the interpreter's state of knowledge at the end of the process. This is not a situation that only applies to a particular class of people in need of extra support. It is a feature of everyday life. Imagine the following unremarkable exchange:

*Zhuangzi:* ‘What do you want to eat?’

*Huizi:* ‘I'm not sure’.

*Zhuangzi:* ‘We haven't had fish for a long time’.

*Huizi:* ‘Oh yes, fish. Perfect’.

Huizi wanted to eat something before this exchange, and he wanted to eat fish after it. The process of interpretation changed his desire. It may be tempting to attempt to avoid this conclusion by imagining that Huizi already had an unconscious desire for fish. Then he always wanted fish, and Zhuangzi, by artful interpretation, led him not only to expressing his preference but also to greater self-knowledge. Fantastical stories of this kind are not innocent. They obscure the agency of the person that they are told about. Huizi's development of a preference for fish is his active response to his environment; but if the desire is written back in time, this is lost. He is, instead, pictured as an unresponsive lump from which Zhuangzi excavates an already-existing preference. Applied to mental disability law, such stories subtly misattribute the person's agency to their supporter. The tale becomes one of an active supporter expertly obtaining a preference from supposedly inactive person with disabilities. This would not accord with the general principles of the UNCRPD. In particular it does not show ‘respect for inherent dignity, individual autonomy including the freedom to make one's own choices, and independence of persons’ (Article 3(a)).

Huizi's sudden desire for fish is probably better categorised as a ‘preference’ than his ‘will’. Even if, however, a person's ‘will’ is characterised as their deeper, more stable, desires and values (Arstein-Kerslake and Flynn, 2016: p. 13; Szmukler, 2017: p. 94), then an analogous point will hold. Like preferences, these more durable features of person start somewhere. No one is born knowing that they wish to pursue a career as an architect or consciously endorsing the particular doctrines of any religion. Often, perhaps even more often than for mere preferences, such life projects emerge in the space where self-interpretation and interpretation by others interact. This support is the stuff of everyday life: the encouraging teacher who helps a pupil to see that they have options self-doubt had stopped them from considering; the child on holiday whose trust for the young adult supervising the pool causes them to consider parenthood for the first time^12^; or the person who sits up all night with their friend who is considering divorce, talking over the options.

This last example, friends discussing whether one should divorce, also illustrates the ways that stories are told that project the will back in time before its historical point of origin, as memories are rewritten to ‘preserve a coherent self-image’ (Bortolotti, 2015: p. 136). If the person decides to divorce, then later they might say ‘it was what I wanted, I just didn't know that I wanted it’ about the time immediately before the discussion with their friend. This might capture the way the final decision reflects long-standing and deeply-held commitments; but if the person began the conversation in genuine doubt and ended it genuinely resolved on a course of action, it also obscures what has changed. Even if the decision to divorce does reflect a long-standing commitment to demanding a certain level of behaviour from a partner, it also marks the end of a long-standing commitment to that particular relationship. In other words, a new will is formed in these situations: a will that the conflicts between other long-standing commitments should be resolved by acting in a particular way.^13^ The person's will emerges from the shared process of interpretation and self-interpretation. Acts of interpretation do not merely assess another person's will. Sometimes, they change it.

If it is admitted that processes of interpretation can change will and preferences, then the epistemic reading must be abandoned. There are both good and bad ways of influencing another person's will and preferences; but a reading that evaluates only the interpreter's state of knowledge cannot evaluate their influence on the person. Domestic or institutional abuse provides the clearest example of this point. When someone shows a certain amount of independence, then a controlling or abusive carer might always ask ‘are you really sure that you want to do that?’ They will not ask this question when the person acts in ways that heighten their dependence on the carer. In the right conditions, especially if the person is heavily dependent for a long period of time, this pattern of questioning may be enough for self-doubt to undermine someone's will to be independent (adapted from Benson, 1994). Indeed, if they are dependent on the supporter from a young age, they may never develop a will for independence in the first place.^14^ Yet all the carer has done is ask an interpretative question, and it is one that probably will allow them to better understand the person's preferences. They might conclude that the person does not wish to be independent, and that conclusion might be true. It might accurately reflect the person's will and preferences. On the epistemic reading it is therefore an act of ‘best interpretation’.

A similar point applies to good interpretative support: the epistemic reading cannot always capture what is good about it. Imagine an interpreter who is careful to obtain the interpreted person's opinion on all the available options in whatever situation it is that they face. Doing so is clearly an act of interpreting the person's will and preferences; but quite often the person will be unaware of an option before the interpreter seeks their opinion on it. The epistemic reading, however, cannot distinguish between an interpreter who presents the person with new options and one who does not bother. Both will have a ‘true’ interpretation of the person's will and preferences. The difference between the two is that the interpreter who explored all the options has given the person an opportunity to change their will and preferences.

### 4.3 Everyday interpretation in the hard cases

The problems with the epistemic reading raised in first two parts of this section might be dismissed as an artefact of looking at everyday interpretation. It might be though that if communication is particularly difficult, then the person will be less able to express their agency during interpretation and will be less likely to change their will and preferences, so the problems with the epistemic reading will be marginal. This subsection gives a number of reasons for resisting this conclusion.

The first reason to reject the epistemic reading even when communication is difficult is that, as shown in subsection 2.3, GC1 is a call to include people facing serious communication difficulties in everyday practices, not a call to develop a special process just for them. If expressing agency and changing preferences are important parts of interpretative practices in daily life, then the aim should be to make them available even to those who have serious problems with communication. In some cases the attempt might fail, but that does not justify not making it.

Second, rather than supporting the epistemic reading, communication difficulties undermine it. Severe communication difficulties make people less able to rebut incorrect presumptions, so interpretation in these circumstances will be less accurate. It makes little sense to reduce ‘best interpretation’ to ‘true interpretation’ only in the cases where it is least likely to be true. Beyond this, limiting the epistemic reading to cases of severe communication difficulties creates a contradiction. It simultaneously asserts (A) that interpretative process do not change the preferences of people with severe communication difficulties and (B) that their preferences cannot known due to the communication problems. If, however, their preferences cannot be known, then the claim that they have not changed cannot be evidenced.

The final reason to avoid the epistemic reading is due to the particular form of reasoning that is often recommended in cases of serious communication problems. It typically takes the form: ‘*P* cannot communicate a preference to us; but if they could communicate, then they would express a preference for *X'* (Flynn and Arstein-Kerslake, 2014: pp. 84–85; Gooding, 2015: p. 55). This is what is known as a counterfactual claim: the first clause, ‘*P* cannot communicate’ denies that something is true; and the rest of the sentence states what would have been the case if it had been true.^15^ There is an obvious contradiction between this form and the epistemic reading, which assumes that all that matters to an interpretation is that it is true. Counterfactuals are explicitly about things that are not true. Furthermore, counterfactual reasoning turns on the idea that some alternative world is more similar to the real world than other alternative worlds, and this central concept of ‘similarity’ is entirely context-specific (Lewis, 1986: pp. 251–252). It requires ideas about what is important, not only ideas about what is true. The processes suggested for the most difficult cases of interpretation are no more compatible with the epistemic reading than other interpretative processes are.

## 5 The substantive reading of ‘best interpretation’

### 5.1 Using the UNCRPD and GC1 to develop a process reading

This section suggests an alternative to the epistemic reading of ‘best interpretation’. On this alternate reading, the accuracy of the final interpretation is an important norm that interpretation should be evaluated against, but it is not the only one. The substance of interpretation must also be evaluated: how it operates, what presumptions it relies on, and what effects it has on the person. It must be evaluated against the full array of rights in the UNCRPD. This subsection shows that it is possible to read GC1 in this way, and the next one addresses three problems that this substantive reading may face in practice. The aim of this section of a whole, however, is merely to show that the substantive reading is plausible, not to develop all of its details.

The substantive reading is possible because the contextual cues in GC1 that have suggest the epistemic reading are ambiguous. The binary between ‘best interests’ and ‘will and preferences’ is largely qualified. It occurs at the level of ‘paradigms’, ‘regimes’, ‘systems’, and ‘primacy’ (paras. 21, 26–29); and this falls short of what the epistemic reading requires. A contrast between, for example, a ‘best interests paradigm’ and a ‘will and preferences paradigm’ does not necessarily imply that a supporter attempting to determine someone's preferences cannot draw on ideas about what might be good for them at all. Nor does it imply that a supporter cannot encourage the person to change their preferences at all. It merely implies that the overall goal of the process is to attend to the person's will and preferences rather than whatever is believed to be their objective good. To put it another way, in GC1 consideration of the person's good can influence the process of interpretation, but it cannot dictate the outcome of that interpretation. For example, the thought that all people need food to survive can cause an interpreter to raise the issue with and challenge a particular person who is not eating. What GC1 would not allow is allowing this objective good to justify ignoring the person's apparent preference or, because it amounts to the same thing, simply concluding that the person ‘really’ wants to eat, no matter what they say and do.

There are only a few places where GC1 directly addresses particular acts of interpretation. Indeed, only three statements can be characterised in this way: it states that ‘the “best interpretation of will and preferences” must replace the “best interests” determinations’ (para. 21); it defines substituted decision-making regimes as including those in which ‘any decision made by a substitute decision-maker is based on what is believed to be in the objective “best interests” of the person concerned, as opposed to being based on the person's own will and preferences’ (para. 27); and it states that ‘all forms of support in the exercise of legal capacity …must be based on the will and preference of the person, not on what is perceived as being in his or her objective best interests’ (para. 29(b)). These statements address individual decisions and contain a binary between determining the person's will and preferences and their best interests. Even these statements, however, do not require the epistemic reading. None of them forbids an interpreter from respectfully influencing a person's preferences as part of the process of ‘best interpretation’.^16^

It is not, however, enough to simply reject the epistemic reading. If truthfulness is important to interpretation, but so are the ways that interpretation can provide an avenue for self-expression and changes in will and preferences, then it is important to have some idea about the norms that distinguish between acceptable and unacceptable influences on the person. The Comment itself can be read as suggesting substantive norms to guide interpretation, and doing so also makes some of its more puzzling passages clearer. In particular, it states that ‘the best interpretation of will and preferences … respects the rights, will and preferences of the individual’ (para. 21). How does interpreting only will and preferences also automatically respect rights? If best interpretation is reduced to true interpretation, this offers a dilemma. It requires believing either that conflicts between rights and ‘will and preferences’ are impossible, despite such situations being a regular feature of both everyday life and the secondary literature (Richardson, 2013: p. 100; Gooding, 2015: p. 61; Szmukler, 2017: p. 94), or believing that preferences, no matter how fleeting or mistaken, must always trump rights, no matter how important, despite the implausibility of that position.^17^

A substantive reading, one that does not reduce ‘best interpretation’ to ‘true interpretation’, avoids these problems. It simply reads the other rights found in the UNCRPD into the word ‘best’. In other words, ‘the best interpretation of will and preferences’ respects ‘rights’ because if the interpretation does not respect the person's rights, then it is not the best interpretation. To use an extreme example, it is possible to obtain a ‘true interpretation’ of a person's preferences by abusing them until they prefer whatever the abuser wants. On a substantive reading of GC1, this is not the ‘best interpretation’ for the simple reason that it breaches Article 16 of the UNCRPD. To give a positive example, on the substantive reading, it is permissible for an interpreter to presume that the person that they are interpreting wishes to have opportunities to take up paid employment on an equal basis as others (Article 27) if, and only if, they attempt to give that person an opportunity to rebut the presumption. On this reading, the UNCRPD itself provides a series of starting points for interpretation, although it is necessary to test those starting points by engaging the person.

This reading can be supported by reference to other parts of GC1. It states that ‘a supported decision-making regime comprises various support options which give primacy to a person's will and preferences and respect human rights norms’ (para. 29). This is not a claim that respect for will and preferences must trump respect for ‘human rights norms’. It is a call to respect both at the same time. Indeed, the Comment expressly relies on particular norms found in the Convention when discussing ‘support in the exercise of legal capacity’, which is described as being ‘based on the will and preference of the person’ (para. 29(b)). For instance, it requires ‘protection against undue influence’ and understands ‘undue influence’ to include cases in which the person is deceived (para. 22, drawing on Article 12(4)). This, however, suggests that interpretation is not simply a matter of accurately determining the person's will and preferences. If those preferences are the result of deception, it will also require the creation of opportunities to change them. Similarly, the Comment requires the provision of information in an accessible manner (para. 17, drawing on Article 12(3)). This is not a matter of discovering what the person prefers as accurately as possible. It is a matter of assisting the person to form the best preferences that they can.^18^ These provisions cannot be accounted for by the epistemic reading. From the substantive reading they are making relatively obvious points.

### 5.2 Practical difficulties for the substantive reading

Although this article merely shows that the substantive reading is plausible, rather than addressing all potential problems with it, three related issues should be addressed at his point: conflicts between different rights; conflicts between a person's rights and an expression of their will and preferences; and conflicts within the person's will and preferences. An example of the former, conflicts between rights, is a person who both wishes to leave an institutional setting in order to live in the community (Article 19) and wishes to maintain their health and ‘physical and mental integrity’ (Articles 17 and 25), when there are good reasons to think that away from the institution they will neglect to take medications and their physical health will deteriorate. The person expresses a preference for both independence and health, and both are rights, so the substantive reading does not seem to offer a solution. Nor should it, if ‘solution’ is taken to mean a single answer appropriate for all cases. Sometimes rights really do conflict. Difficult moral dilemmas are an inescapable part of human life, and the UNCRPD aims to include people with disabilities in humanity's shared difficulties, not to somehow save them from the inconvenience (Preamble (a), (c), (e), (g), (k), (m), (o); Articles 3, 9, 19, 26, 27, 29, 30). The substantive reading, however, requires attention to conflicting rights during the interpretation of the person's will and preferences, and that attention may suggest new avenues that will resolve the conflict for that particular person. In the example, the person may agree to a daily visitor who reminds them to take their medication.^19^ Pragmatic solutions like this are not new, but they resolve a particular instance of conflicting rights. The substantive reading admits that no single ideal, such as freedom, can guide practice. Instead it responds to ‘a whole plantation of such ideals which will all need to be balanced against one another’ (Midgley 2017: p. viii).

The second issue, a person's expression of their will and preferences conflicting with one of their rights, more directly implicates the substantive reading. To put the core issue in its bluntest form, does the substantive reading ever allow a person's expression of will and preferences to be disregarded in favour of the protection of one of their rights? The answer follows from a series of points made earlier in this article. On the substantive reading, ‘best interpretation’ is a process, not merely the outcome of that process. This process of interpretation, as shown in section 4, must start with some interpretative presumptions, and should provide the interpreted person with opportunities to express their agency and change their will and preferences. The substantive reading makes all of the rights found in the UNCRPD into interpretative presumptions; but another common and important interpretative presumption is that people generally mean what they say. This class of cases are therefore reducible to a clash of interpretative presumptions. When the person first expresses a preference that may conflict with one of their rights, however, this conflict of presumptions has not yet been resolved. Nor has the process of best interpretation, which should allow them to change their views, occurred. From these points, an approach to conflicts between rights and expressed will and preferences follows. The substantive reading allows the person's rights to be favoured over their currently expressed preferences if, and only if, one of two conditions exists: (A) there are realistic grounds to believe that a non-coercive (GC1 paras. 22, 27(ii)) process of interpretation and support might change the person's will and preferences; or (B) there is genuine doubt about how to interpret the expression of will and preferences. An example may make the approach clearer.

Iris has recurrent depressive disorder with psychotic symptoms during some episodes. When she is depressed, she sometimes experiences delusions, such as the belief that people attempting to help her must have ulterior motives because she is ‘no good'. She has attempted suicide during previous episodes, but for the last few years she has managed her condition with medication and by self-referring to services when she feels that she is ‘spiralling’ into depression. On this occasion, however, contact with services is initially made by her brother. He reports that Iris is ‘very poorly’, stopped taking her medication about a week ago, and has said things that indicate that she is seriously considering suicide. When the community crisis team visit Iris, she expresses a wish to die and refuses all assistance, saying to the nurses that they are ‘only worried about their own jobs'.

This example contains a conflict between Iris's expressed preference and her right to life (Article 10). It is important to note, however, that on the substantive reading it does not yet contain a conflict between the best interpretation of her will and preferences and her right to life. The presumption that a person means what they say counts in favour of taking her expressed wish to die at face value. At the same time, however, the presumption that generally people wish to live and Iris's history of sometimes wishing to die but generally acting to overcome that wish both count in favour treating any presumption that her expressed wish articulates all of her ‘will and preferences’ with great caution. Furthermore, based on her history, there are realistic grounds to think that with support she may change preferences. After all, she has done so before. Although this example is just an outline and the substantive reading is necessarily responsive to small changes in the facts, it would in this case require seeking to persuade Iris to engage with support and ultimately change her mind. It would not justify simply allowing her expressed wish to die to override all other concerns.

Iris's suspicion of professionals raises another question. If she refuses to engage with the processes of best interpretation and if her threat to her own life is imminent, then might it be permissible to coerce her? If the underlying legal basis for coercion also applies when those without disabilities appear to be presenting an imminent and serious threat to themselves,^20^ then a substantive reading will permit coercion in variations of the same two conditions as it permits favouring the person's rights over their expressed wishes: (A) there are realistic grounds to believe that after the initial coercion, the person will engage in a non-coercive process of interpretation and support and that this process might change the person's will and preferences; or (B) there is genuine doubt about how to interpret the expression of will and preferences, and after the coercion it will realistically be possible to resolve this doubt.^21^ If there is no doubt about the person's will and preferences and no realistic prospect of the person subsequently engaging in non-coercive processes that may change their will and preferences, then on the substantive reading there is no basis for coercion.

It is necessary to distinguish conflicts between a person's expressed will and preferences and their rights from conflicts within their will and preferences. Any account of interpretation should allow for the possibility of the latter. It is important to allow the ‘best interpretation’ of a person to recognize that they may be, as we all sometimes are, ‘unfinished and fragmented' (Jaspers 1997: p. 760). This possibility is, however, foreclosed in some recent accounts of interpretation. For instance, it is thought that a particular act of interpretation might be justified by reference to what the person ‘authentically’ wants (Szmukler and Bach, 2015: p. 7), their ‘will’ as opposed to their ‘preferences' (Szmukler, 2017: p. 94), the person's ‘narrative’ (Bach and Kerzner, 2010: pp. 63–67), or their ‘values' (for a critical account, see Banner, 2013: pp. 75–78). All such accounts posit a higher, unified self – the authentic self, the self that has a coherent narrative, or the self with integrated central values – that transcends the messy and contradictory empirical human being. These theories face fundamental problems with both in theory (Garnett, 2017) and in application to decision-making law (Burch, 2017); and, as Midgley observes, when they are mistaken for the whole of morality, they quickly become ‘incoherent’ (1991/2017: p. 104). In the current context, however, the particular danger of these theories is that they treat two morally different situations in an identical manner; and, although in one of these situations they are harmless, in the other they are misleading. Two examples will make this point clearer.

The harmless use of these theories is when the process of interpretation allows the person to resolve a conflict in their will and preferences. For example, imagine Kirsty knows that when she drinks, she ‘loses control’ and consequences follow that she prefers to avoid; but she also wants to go for a beer with old friends. If, with a supporter, she reaches the conclusion that even one beer is not worth the risk, then there is no harm in saying that she ‘authentically’ wants to avoid drinking on this occasion. Such cases should be distinguished from ones in which it is the interpreter, not the person, who resolves a conflict between different preferences. Consider the following example:

Gary has a severe learning disability and, following a traumatic childhood experience, is terrified of hospitals and doctors. Gary enjoys his life, and any process of interpretation would conclude that he wants to live; but he has contracted a chest infection that the community nurse strongly suspects has developed into pneumonia. The only way to get a chest X-ray is in the local hospital; and the likely treatment, high-flow oxygen therapy and intravenous antibiotics, can only be administered in hospital.

In these circumstances, even with all practicable supports, Gary might not be able to resolve a conflict between his will to live and his will to avoid hospitals. His fear might stop him from being able to consider hospitals at all, and his learning disability may prevent him from abstractly reflecting on his own fear. For practical reasons, an interpreter may have to choose one will or another; but, if so, they must be clear that it is them, not Gary, who is doing so. It is in cases like this that ideas of ‘authenticity’ and ‘narrative’ are dangerous. Kierkegaard (1843/2008: JJ:167), characteristically, sums up the underlying point in an aphorism: ‘it is quite true what philosophy says, that life must be understood backward. But then one forgets the other principle, that it must be *lived forward*’. The past and the future are not, for living creatures, symmetrical; and appealing only to a person's ‘narrative’ does not help when the question is what their narrative should be. As Lippitt (2007: p. 58) writes, pretending otherwise risks ‘over-simplifying what a human life is like’. When it comes to ‘best interpretation’ to claim that the interpreter's resolution of a conflict somehow embodies the person's values, authenticity, or narrative is to favour an imaginary unified self, ‘an empty puppet’ (Harmon 1990: p. 69), over the empirical conflicted person. Instead, the substantive reading should require an interpreter to openly admit that is they who are reaching a final conclusion based on what they know of the person's conflicted will and preferences and the rights found in the UNCRPD.

## 6 Conclusion

This article makes two central points. First, the epistemic reading of ‘best interpretation of will and preferences’, one which reduces the word ‘best’ to the word ‘true’, should be avoided. Interpretative acts are complex, and can change a person's will and preferences in ways that can be either good or bad. Some way of evaluating these diverse effects, which the epistemic reading cannot provide, is needed. Second, the problem with the epistemic reading is not necessarily a problem with GC1. The substantive reading, guided by the rights found in the UNCRPD, is consistent with the Comment and avoids the problems that the epistemic reading faces. Although the purpose of this article has been to give a particular account of best interpretation rather than to compare it to the best interests standard, it may have implications for that wider debate. In particular, it suggests that any attempt to compare the two should first be clear about which reading of ‘best interpretation’ is being used and why.

## Funding

This research has been conducted as part of the Mental Health and Justice multi-disciplinary research initiative, funded by the Wellcome Trust. The funding source had no involvement in the design, content, or writing of this article and no involvement in the decision to submit.

## Footnotes

1 Convention on the Rights of Persons with Disabilities (adopted 13 December 2006, entered into force 3 May 2008) 2515 UNTS 3, Article 12.

2 These observations are not legally binding, but States that disagree must ‘engage …and present good counter-arguments’ (Martin et al. 2016, p. 56).

3 The contextual remark, ‘after significant efforts have been made’, does not settle this ambiguity. ‘Significant efforts’ may be either attempts to begin a process or part of a failed process.

4 Although even at the individual level, this may be because the ‘best’ of ‘best process’ and ‘best outcome’ circularly redefine one another.

5 This distinction between overall and particular will and preferences can, however, raise other problems. See Burch (2017).

6 These last two examples also illustrate a danger with any attempts to legislate too closely for ‘correct’ interpretation. The law tends towards literal-mindedness, but irony is so prevalent and powerful in everyday communication that it is arguably ‘constitutive of human excellence’ (Lear, 2011: p. 37).

7 This alone is enough to characterise a system as a substituted decision-making regime (UN Committee on the Rights of Persons with Disabilities, 2018).

8 This influence is discussed in Arstein-Kerslake and Flynn (2016: p. 472).

9 Arguably, however, Szmukler is not careful enough with the particular difficulties caused by the normativity of interpretation. For instance, he treats holding anyone with a belief or value to a normative commitment not to simultaneously assert the opposite as unproblematic. This raises two serious problems. First, the generalisation of the principle of non-contradiction from beliefs to values and desires is deeply suspect. To say, for instance, ‘I love him, but I don't love him’, is more than simply intelligible. It describes a mixture of passion and ambivalence so common that it is a cliché. Second, even in logic, the place where the principle of non-contradiction might be thought safest, it is contested (Priest, 2006). As a consequence, Szmukler's claim that non-contradiction is a universal value that ‘cuts across cultures’ is far too strong, but any weaker form of it fails to serve his purposes: if it only matters sometimes, then what norms govern its use?

10 There is no need for someone making a presumption to consciously think of it in every case. As Anscombe (2000: §42) writes, there is a difference between ‘an order which is there whenever actions are done with intentions’ and ‘actual mental processes’.

11 This form excludes so called ‘irrefutable presumptions’, which are better described as ‘stipulations’ (Rescher. 2006: p. 5).

12 Note how little expertise an interpreter needs to begin a process that changes someone's will. Children do it.

13 In terms of Frankfurt's (1971: p. 10) influential paper, the person may have begun the conversation with a desire to divorce or even a second-order desire to want to divorce, but they ended it with a second-order *volition* to divorce: a desire for the desire to divorce to effectively guide their actions.

14 This is an issue that sometimes arises in the Court of Protection of England and Wales. For instance: *A Local Authority v M* [2014] EWCOP 33, [2015] COPLR 6 [230].

15 As Lewis (1973: p. 3) points out, without the first clause, ‘if they could communicate’ would imply that they cannot communicate as a matter of typical English usage, but it would not necessarily do so as a matter of logic.

16 Beyond this, indirect evidence that GC1 does not treat ‘best interests’ and ‘will and preferences’ as entirely irreconcilable opposites emerges in its discussion of children with disabilities. As the UNCRPD requires, it allows best interests to be the primary consideration if the child's will and preferences are also ‘respected on an equal basis with other children’ (para 36).

17 Even Mill (1859/2003: p. 158) thought that someone's right to life would have priority over their preference to cross a bridge that they did not know was unsafe. He, admittedly, claims that the basis for intervention is the person's desire to live. This justification is, however, of entirely the wrong form. It assumes that effect is being given to a single unitary desire, to live; but, on his own telling, the example contains a conflict between two desires, to live and to cross the bridge. The person crossing the bridge does not desire to resolve the conflict in this particular way because, again on Mill's own telling, they are unaware of the conflict. Basing a decision on the idea that the person would resolve the conflict in any particular way, if only they had more information, is to privilege an imaginary alternative person over the existing one. This necessarily draws on norms other than truth and so abandons the epistemic view: see the discussion of counterfactuals at the end of the previous section and Harmon (1990).

18 A similar point applies to the requirement for medical treatment to be based on informed consent (2014: paras. 41–42, drawing on Articles 14–17 and 25).

19 If this support is not available, then the State may be in breach of the Articles in question (here, 17 and 25) read in conjunction with Article 5, which requires ‘appropriate steps to ensure that reasonable accommodation is provided’. For conceptual clarity, it is necessary to address this issue *before* the question of ‘best interpretation’. If the support is not required by the Convention, then failing to provide it does not cause a conflict of rights.

20 See Articles 12(2), 14, 15, and 25(d).

21 Flynn and Arstein-Kerslake, 2014, Flynn and Arstein-Kerslake, 2017 appear to recognize the second, but not the first, of these conditions–. This passage merely addresses the acceptability of coercion from the perspective of the substantive reading. For a variety of reasons, it is not a recommendation for a legislative programme: other parts of the UNCRPD apply further constraints to the use of coercion; analysis of best interpretation does not provide guidance on the procedural checks that are necessary on any coercive power; and analysis of best interpretation does not address all of the relevant moral or political issues.
